# Horse Husbandry–Nutrition, Management and Welfare

**DOI:** 10.3390/ani13010169

**Published:** 2023-01-01

**Authors:** Glenys K. Noble

**Affiliations:** School of Agricultural, Environmental and Veterinary Sciences, Charles Sturt University, Wagga Wagga, NSW 2650, Australia; gnoble@csu.edu.au

Nutrition is the foundation of health and welfare, going hand in hand with horse husbandry. Without appropriate nutrition, combined with sound husbandry practices, all equine body systems are compromised and thus the health and welfare of the horse are negatively impacted. It is then unrealistic to expect this compromised horse to perform at its optimal level and/or behave according to human expectations, expectations that can potentially compromising health and welfare even further.

So, how can sound welfare be accomplished—best practice or best fit? The concept of best practice and best fit come from human resource management. Best practice implies the existence of one optimal method of achieving a highly effective or desired outcome. Best fit assumes that the relationship between desired outcomes and management practices is not linear but is mediated and/or moderated by contingency variables, such as available resources, knowledge and/or experience level and constraints. The focus should be on achieving the most desirable fit between relevant factors to achieve the optimal outcome [1].

Around the world, horses are maintained for many different purposes and in many different conditions. There are many countries where horses are still largely working animals, remaining essential for the generation of income and improvement of the daily lives of their owners. Poor horse welfare, additionally, has human impacts in cases where an animal is unable to work to its full potential [2].

In westernised countries, horses are utilised largely for leisure activities with horse racing being readily identified as a major industry. This industry is often challenged by the general public on issues of welfare arising for horses being highly trained for racing [3]. Identifying key welfare issues facing racehorses should assist in improving welfare standards, but there is still a lack of scientific evidence on what the key issues are and their relative importance [3].

Like humans, horses are living longer. This longevity often comes with age-associated health conditions, such as arthritis. It is imperative that this group of horses is appropriately managed to ensure good welfare outcomes. With welfare being defined as an animal’s ability to cope with the environment it finds itself in [4], good welfare implies that the horse is coping well in its given environment. The challenge is to measure objectively how well a horse is living under different housing and management conditions. Little has been published on the impacts of different management strategies on horse health and welfare, but horse managers are often criticised for not making evidence-based decisions, instead relying on experience, common sense and anecdotal beliefs [5].

It does appear, however, that when ancient practices are compared to modern horse husbandry there are many similarities. This is especially the case in terms of breeding strategies, herd management, feeding and training approaches. Certainly, horses have been considered high-value animals since the Iron Age. Due to this high value, they were seen to require special expertise in their care [6].

While the general rules of horse husbandry may not have changed much, there is the potential for the application of novel solutions to horse husbandry to enhance welfare. The use of pheromones to assist in calming horses during routine procedures, similar to pheromone therapy used to placate nervous dogs, is one such avenue [7].

This Special Issue, entitled “Horse Husbandry: Nutrition, Management and Welfare”, presents a review delving into ancient horse husbandry practices [6]. This is accompanied by five research papers presenting the latest findings on several aspects of horse management and welfare. The collected papers address how well several classes of horse, including aged horses with chronic health conditions, adapt to a variety of housing conditions. This determination is made using objective measures such as hair cortisol concentrations [5] and time budgets [6]. The welfare status and care of two very different groups of horses, working horses in Honduras [2] and racing Thoroughbreds in Australia [3] is presented, demonstrating the importance of sound welfare in both situations. Despite the two groups of horses seemingly being worlds apart, there are striking similarities in what is considered important for the care and management of these two groups. Finally, a novel non-invasive approach, using pheromones to assist horses during routine procedures that cause discomfort or act as stressors, was evaluated [7].

The review by Klecel and Martynuik [6] on the early development of horse breeding and management demonstrated that many ancient horse husbandry and breeding practices are still used to good effect today. The horse was domesticated initially as a source of meat and milk, albeit much later than other livestock such as cattle, sheep and goats. The utility of the horse led to its use in transportation, which persists to the present day, as well as in warfare. It was the horse’s use in warfare that directly led to its use for racing. The enjoyment of the horse in racing has existed since the Iron Age. The scale of horse racing in the Iron Age was comparable to today’s racing in Great Britain and France, with its role in society even more significant than that it enjoys today. Furthermore, the Greeks and Romans were aware of the importance of factors such as rider/driver weight carried by the racehorse, as well as their skills, the significance of which is still being researched today.

The relevance of Greek and Roman horse husbandry practices remains high in the modern era. While feeding and training systems have undergone modernization, some similarities with historic principles can be found. The ancient diets included a limited portion of grass and legume plants, cereals such as barley (sometimes boiled), and pasture grazing at specific periods of the day or night [6].

High protein/energy feed most commonly used in the Roman Empire seems to be barley and unidentified “beans”, i.e., legumes. Ancient horse managers also recognized differences in nutritional demands between specific groups in the herd, now called classes of horse. For example, there was an additional ration of barley for mares with foals or a particular “mixed forage” for youngsters undergoing the first stage of training [6]. In contrast to modern practices, water access was limited to once a day or less, although the importance of electrolytes was apparently understood, with horses given salted or malt water after strenuous exercise.

The authors concluded that horse breeding and management are the result of the continuous evolution of practices. It is noted that in the context of five millennia of horse domestication there have been slow but steady improvements. The importance of horses, both then and now, is clear from the investment made in their development and the enhancement of the scope of their uses.

Horses are kept under a wide variety of husbandry conditions, influenced by a plethora of factors, and the focus of horse welfare research has long been to determine the ideal living conditions for horses. Two research papers investigated the effect of different housing conditions on horse health and welfare using two, quite different objective measures.

Mazzola et al. [5] measured hair cortisol concentrations in forty-seven adult horses of mixed age and breed to assess long-term stress under three different housing conditions. Hair samples were taken from all horses on the same day at four time points of the year (summer, autumn, winter, spring).

Group One (*n* = 12) were individually stabled at night, with access to a paddock with conspecifics during the day. Horse diets were individualised according to needs and included, in addition to paddock grass and hay placed in the racks, a supplement of concentrated feed given morning and evening in the stables. Horses were routinely dewormed and underwent periodic hoof care as well as prophylactic vaccinations.

Group Two (*n* = 19) were paddocked day and night in permanent social groups. The paddocks had large sheds where horses could shelter during unfavourable weather conditions, as well as automatic waterers. Horses’ diets were managed like those of Group One— on the basis of individual needs. A supplement of concentrated feed was given in the evening in separate feeders. Additionally, paddock grass and hay were placed in hay racks. Routine health and health care was as for Group One.

Group Three (*n* = 16) lived free in a herd on permanent grassland with trees for shelter, natural water sources and grasses to eat. No supplementary feeding was provided nor any medical treatment including deworming and vaccinations. No horse received any hoof care.

Independently of the management strategies deployed, significantly higher hair cortisol concentrations were found in the summer and autumn, as well as in horses older than 15 years. In summer, autumn and winter, hair cortisol concentrations were significantly lower in Group One than Group Two, indicating that Group One horses had better homeostasis. Group Three had intermediate hair cortisol concentrations between the other two groups in all seasons. It appeared that spending the night in the stable had a positive impact on the well-being of these horses. The authors suggested that this could be related to sleep quality, as sleep is an essential facilitator of physical well-being, given that horses sleep for an average of only 3 h a day. It was suggested that the elevated hair cortisol concentrations in older horses were related to a lower ability of aged horses to comply with stressors, and hence older horses should be even more carefully managed.

Keleman et al. [4] investigated the equine activity time budgets of geriatric horses. Test subjects were both with and without chronic orthopaedic disease and were managed under several different management systems. Using the Hoofstep^®^ automated equine monitoring system, the time budgets of 104 horses (51 mares, 53 geldings, aged from 2 to 32 years) of mixed breeds under three different management systems and daily routines, each assigned to one of four the health/age groups, were assessed.

The three housing systems were arranged as follows. Horses were housed in individual box stalls with a paddock turnout (Stable); small groups (2–3 horses) were kept in stalls attached to yards and paddock turn out (Paddock); and the open-air group (9–10 horses) was housed in a paddock with a run-in shelter (Pasture). Horses had ad libitum access to clean water and were fed ad libitum hay during paddock turn-out, with additional hay being placed in the stable in the afternoon. Horses with poor dentition or additional nutritional requirements received supplementary feeding with hay pellets.

The health/age groups were: (1) younger than 20 years with chronic orthopaedic diseases (*n* = 45); (2) geriatric horses ≥ 20 years with chronic orthopaedic disease (*n* = 42); (3) sound (lameness < 1) geriatric horses (*n* = 7) and (4) sound horses younger than 20 years (control group, *n* = 10).

The Hoofstep^®^ monitor measured four main behaviours: (1) “feeding” (the time the horse is chewing—in any position or combination with other behaviours), (2) “resting” (without distinguishing between lying and standing), (3) “active” (slow locomotion, walk) and (4) “highly active” (fast movement (trot, canter) and potential stress behaviours such as headshaking). Horses were monitored twice each for 5 to 10 days in spring/summer and autumn/winter.

The findings of this research were similar to those coming from other time budget studies. Regardless of health/age status, horses kept in paddocks and at pasture spent significantly more time eating than horses housed in stables. Horses housed in stables spent significantly more time resting than those housed in paddocks and at pasture, with horses at pasture spending the least time resting. Horses in paddocks spent significantly less time engaged in slower movement than stabled horses, with horses in pasture spending the most time engaged in slow movement. Unsurprisingly, there was a significantly higher level of fast movement for the group of horses kept at pasture compared to those in either paddock or stable, with stabled horses carrying out the least amount of fast movement [4].

The authors found that horses in this study were eating for 42% of their day. This is within the wide range of 10–64%, measured for domestic horses, but also below the 50.82–66.6% reported for semi-feral horses. Eating times were highest in the morning and the afternoon and lowest in the night and very early morning hours. This was even the case in horses that were turned out on pasture overnight during high summer temperatures. The overall time budget for resting of 39%, which included periods of inactivity and sleep, was higher compared to free-ranging conspecifics [4].

Surprisingly, the geriatric horses, as well as horses suffering from chronic orthopaedic disease, had time budgets equivalent to the healthy control group and were mostly within the ranges observed in free-ranging conspecifics. The results show that being in appropriate living conditions age and/or orthopaedic disease does not significantly affect equine behaviour time budgets. While similar time budgets do not imply good welfare per se, they do indicate an equal ability of the geriatric and chronically lame horses to cope with their environment in comparison to the healthy control group [4].

In the present study, horses living at pasture had a more uniform temporal distribution of feeding and movement. Specifically, less pronounced peaks were seen compared to the analysis of horses living in more restricted husbandry systems (stable and paddock) [4]. This may be associated with less stress and better accommodation of equine gastrointestinal physiology, thus contributing to horse welfare. Welfare is defined as an animal’s ability to cope with the environment it finds itself in. Welfare optimization strategies should be based on the establishment of highly motivated behaviours and corresponding time budgets that contribute to equine well-being in their specific environment, rather than blindly pursuing wild-like behaviour as the gold standard. This indicates that best fit, rather than best practice, is the way forward [4].

Two studies investigated human aspects of equine welfare in two vastly different contexts. Wild et al. [2] investigated owners’ knowledge and approaches to colic in working equids in Honduras, while Mactaggart et al. [3] aimed to identify the key welfare issues of Thoroughbred racehorses in Australia by industry stakeholders.

The aim of the Honduras study was to define the role and improve the welfare of working equids through obtaining a baseline understanding of equine colic or ‘abdominal pain’. The objectives were: to explore owners’ current understanding of colic in Honduras and to identify knowledge gaps where educational materials could benefit owners and working equid welfare [2].

The most common tasks undertaken by equids were firewood collection, transportation, and carrying crops, highlighting their vital importance to local communities and economies. There were variations in the equids’ access to water and roughage, with some experiencing seasonal changes in feeding patterns. Generally, owner knowledge around colic signs, causes, risk factors and treatment was found to be lacking, with nearly half of owners unable to name a cause. Most owners with previous experience of colic had treated it themselves, typically using their own herbal remedies [2].

Following the use of their own treatments, agrovets (‘agrovets’ sell veterinary pharmaceuticals alongside various other farming tools and equipment) were often the next source of information and treatment for owners. This was the case due to the difficulty in accessing qualified veterinary practitioners is difficult.

Only a small number of colic cases (6%), located near an equine hospital, were treated by veterinary practitioners. Unfortunately, this is not an option for the more remote areas, and so educational resources are clearly needed. The authors recommend that, in many cases, remedies readily available in communities should not be discouraged without reason so long as equid welfare is not compromised [2]—a case for determining and then using best fit, rather than best practice.

Despite some groups of horses having ample access to ‘state of the art’ veterinary care, welfare issues still arise. Thoroughbred racehorses also face compromised welfare due to being highly trained and transported regularly to race meetings. However, there is little documented information on the most serious welfare issues confronting racehorses [3]. A panel of experts working in the Thoroughbred racing industry determined the key welfare issues that were prioritised using a wider stakeholder survey. The panel comprised breeders, owners, veterinarians, sales personnel, farrier, horse transporter, trainer, racing official and a retrainer. Using a facilitator who described welfare indices and their application, industry experts complied the following key husbandry issues that emerged from their discussions: horsemanship, weaning, stabling, environment, heat and humidity, ventilation, nutrition, transport, wastage, gear, track design and surface, health and disease, education of the horse, and whips. For each key issue, up to four levels of husbandry provision were identified. The first of the levels represented the ideal husbandry situation for racehorse welfare, which we might term ‘best practice’, and the last represented the least desirable husbandry option [3].

These issues and levels were used to develop a stakeholder survey for distribution widely across the Australian racing industry. The survey aimed to quantify opinions on the most appropriate levels of each issue. Stakeholder preference was analysed to assess the impact of specific features on welfare to determine which combination of a limited number of levels of issues presented concurrently was most preferred (Best Fit?).

The online survey was emailed to 1773 stakeholders who comprised of the same professions as the expert panel: 315 breeders, 793 trainers, 135 veterinarians, 37 transporters, 119 farriers, 93 sales, 245 racing administrators, and 10 re-trainers with a response rate of 13%. The issue importance levels, ranked from most to least important, were: horsemanship > health and disease > education of the horse > track design and surface > ventilation > stabling > weaning > transport > nutrition > wastage > heat and humidity > whips > environment > gear. No significant effects of stakeholder group were registered in terms of issue importance levels, except for the issues weaning and nutrition, which tended to be rated as less important by the re-trainers, farriers, and veterinarians (nutrition only). Respondents with little experience of Thoroughbred racehorses tended to rate the horses’ environment and gear as more important, whereas those with longer experience tended to rate horsemanship, education of the horse, transport and nutrition as more important [3].

The importance of qualified staff was given the highest ranking by the stakeholders, particularly by those with the greatest experience of dealing with Thoroughbred racehorses. The human–horse relationship is based on a good knowledge of husbandry, skilled handling, care treatment, and an affinity and empathy with animals, dedication and patience [3].

Health and disease were ranked second. This is probably because training and management methods frequently cause physiological problems, which contribute to a deterioration in health as well as the immune system. Housing, exercise, and feeding of thoroughbred racehorses differ from the social structure, behavioural ecology, and activity budgets of feral and free ranging horses, producing performance-related clinical problems because of intensive management, stable design, nutrition, training methods, and transportation [3].

The normal behaviour of the horse is dramatically altered when stabled; social grouping is absent, exercise is limited, foraging does not occur, feed is concentrated and time budgets are altered so that boredom is a frequent outcome. Stereotypic behaviours are commonplace in such instances, believed by many to be coping mechanisms to environmental stressors [3].

The restricted environment of the stables severely reduces the normal pattern of locomotion.

Free-ranging or feral horses take many thousands of paces per day when grazing. Thus, stabling of Thoroughbred racehorses and the feeding of a high-grain ration can cause many welfare problems. This is particularly the case with, enforced immobility followed by intense physical activity [3].

Diet at weaning may also have far reaching effects: grain supplements fed prior to weaning contribute to the development of stereotypic behaviours, supporting a relationship between diet and oral stereotypy in adult stabled horses.

The digestive system of the Thoroughbred racehorse can also cause welfare problems in the intensively managed stabled racehorse with decreased gastrointestinal function, which often results gastric ulcers, colic and laminitis. Feral or free ranging horses spend between 50% and 70% of their day grazing, while racehorses are fed mainly high-energy concentrated grain diets, which may take only 15% of their time to consume. Reduced gastrointestinal function leads to painful conditions such as colic, laminitis, and gastric ulcers, inducing stereotypic behaviour in stabled Thoroughbred racehorses [3].

The authors state that welfare problems stemming from poor nutrition are not universal, thus explaining its middle ranking in the survey by respondents. However, this brings into question the understanding of the term nutrition and the lack of recognition for the totality of nutrition. The authors identified several welfare issues in the areas of health and disease, stabling and weaning that are directly related to nutrition. These include differences in time budgets and behavioural ecology; lack of forage and associated foraging behaviour contributing to boredom, lack of locomotion and the development of stereotypies in both weanlings and adult horses; and feeding concentrates which lead to gastric ulcers, colic and laminitis. Equine nutrition is more than simply the provision of appropriate amounts of nutrients.

It is alarming that veterinarians rate nutrition as a less important issue for the welfare of the Thoroughbred racehorse. This suggests a gap in veterinary education [8,9], rather than veterinarians not being asked for nutritional advice. Veterinarians are identified as a primary source of equine nutritional information [10]. The number of independent equine nutritionists in Australia is small, with most equine nutritionists being employed by feed companies whose goal is to the increase sales of processed feed products. It is reassuring that more experienced respondents rated the importance of nutrition as much higher than those less experienced. Furthermore, women rated nutrition more highly than men. With so many more women entering the racing industry, this superior understanding is a very positive sign.

Choi and Yoon [7] investigated the use of the pheromone Androstenone, derived from boar saliva, that causes dogs to become less excited. Plasma serotonin, β-endorphin and cortisol from a series of blood sampling by venepuncture was measured in a cross-over study using eight horses. The minor pain caused by the repeated blood collection appeared to induce sufficient uneasiness and pain in the horses to decrease plasma serotonin and elevate β-endorphin concentrations in the control group. When horses had administered Androstenone to their nasal cavity, plasma concentrations of serotonin and β-endorphin during blood sampling remained unchanged. The triggering of constant serotonin secretion may result in horses being more tolerant of discomfort. As such, thus Androstenone could be used as an effective aid to calm horses confronted by adverse stimuli [7].

The papers presented in this Special Issue provide thought-provoking insights into many areas of horse husbandry, nutrition and welfare. Horse husbandry practices, aimed at enhancing welfare, certainly constitute a continuous evolution of practice in which there continues to be slow but steady improvements. Pheromone therapy may be one such improvement to assist horses to cope with the discomfort of routine procedures, making use of the measurements of hair cortisol concentrations and time budgets using electronic technology, enabling objective monitoring of horse well-being. While it is evident that aged horses need more careful management to ensure their welfare, it is also evident that older horses and chronically lame horses can cope well with their environment. While the healthcare of working horses in developing countries continues to be a welfare issue due to a deficiency amongst veterinarians, the education of owners, together with the use of appropriate remedies readily available in communities, can bridge this gap to ensure beneficial outcomes for both horses and the people who rely on them. The identification and prioritization of key welfare issues by industrial stakeholders may lead to an improvement in the standard of horse husbandry, not only in the Thoroughbred racing industry but the horse industry in general. While there was some consensus on the ranking of importance, some clear discrepancies were identified for weaning practices and nutrition that warrant further examination. This is particularly important to enable equine veterinarians and retrainers to reduce the inherent welfare risks for retired racehorses entering a second career. Given the breadth and depth of the research presented in this Special Issue, it is clear realized that there can be no one optimal means way of ensuring good welfare for horses. Rather, the best fit should be used to work with choices and constraints to ensure good welfare outcomes.
